# Nest parasitism, promiscuity, and relatedness among wood ducks

**DOI:** 10.1371/journal.pone.0257105

**Published:** 2021-12-02

**Authors:** Kayla Harvey, Philip Lavretsky, Justyn Foth, Christopher K. Williams

**Affiliations:** 1 Department of Environmental and Forest Biology, State University of New York College of Environmental Science and Forestry, Syracuse, New York, United States of America; 2 Delaware Department of Natural Resources and Environmental Control–Division of Fish & Wildlife, Dover, Delaware, United States of America; 3 Department of Biological Sciences, University of Texas at El Paso, El Paso, Texas, United States of America; 4 Division of Bird Habitat Conservation, United States Fish and Wildlife Service, Falls Church, Virginia, United States of America; 5 Department of Entomology and Wildlife Ecology, University of Delaware, Newark, Delaware, United States of America; Texas A&M University College of Veterinary Medicine, UNITED STATES

## Abstract

Nest parasitism is a common reproductive strategy used by many species of cavity nesting birds. Among these, the wood duck (*Aix sponsa*) is known to have evolved very specific strategies of when and whom to parasitize that is often based on population and/or environmental queues. Here, we investigated the genetic relationship of two female wood ducks competing over an artificial nesting box in Delaware, including the continued incubation of one female despite the death and body remains of the other female throughout the incubation process. We test whether such an extreme case of nest parasitism can be explained by relatedness, egg lineage composition, or a combination of other factors. To do so, we extracted genomic DNA from blood and tissue of the females, as well as chorioallantoic membranes of all viable and inviable eggs. Subsequently, we assessed relatedness among females and eggs based on hundreds of nuclear loci and the mitochondrial control region. We concluded that (1) the two incubating females were entirely unrelated, (2) the single clutch is in fact represented by a minimum of four unrelated females, and (3) a single female can lay eggs sired by different males. The latter finding is the first direct evidence for successful extra-pair copulation in wood ducks. With decreasing costs and increasing effectiveness, genomic methods have the potential to provide important insights into more complex ecological and evolutionary tactics of such populations.

## Introduction

Avian nest or brood parasitism is a common reproductive strategy utilized by many bird species, and in particular by waterfowl (Anatidae; [1–3]). Several hypotheses have been proposed to explain the evolution of such reproductive strategies [1–2, 4, 5], including (1) competition for desired nest sites leading to increased parasitism [6]; (2) increasing a female’s reproductive success without incurring the costs of parental care [7]; and (3) maintaining the opportunity for reproduction even if a female is unable to find a suitable nest site or if their personal nest fails [4, 8]. However, it is known that not all females of a population will annually engage, if at all in their lifetime, in some form of parasitism [7]. Thus, understanding why some females more readily utilize this secondary reproductive strategy can shed light into the cause(s) for this evolutionary life-history strategy.

Among North American waterfowl, intraspecific nest parasitism is especially common for tree-nesting species [4] including the wood duck (*Aix sponsa*) in which a large proportion of a population’s females will readily dump eggs among neighboring nests [9]. In addition to nest parasitism, numerous studies have documented wood ducks exhibiting strong nest site philopatry, where females commonly return to the same nesting region and often nest in the same cavity as previous years [10, 11]. Competition can be particularly intense when females attempt to use the same nest that they were successful in previous years. For example, Semel and Sherman [7] documented wood duck females laying eggs in already occupied nest boxes that they themselves used in the previous years. The authors found that the later arriving and competing female(s) would typically often be forced out after a fight. While parasitism has been found to be random in some populations [7, 12], other studies have shown those females that successfully parasitize nests are more likely to do so in nests of related individuals–and perhaps explaining why the parasitized female would accept and raise young that are not her own [13]. Therefore, the amount and probability of particular nests to be parasitized appears to be population-specific.

**Fig 1:**
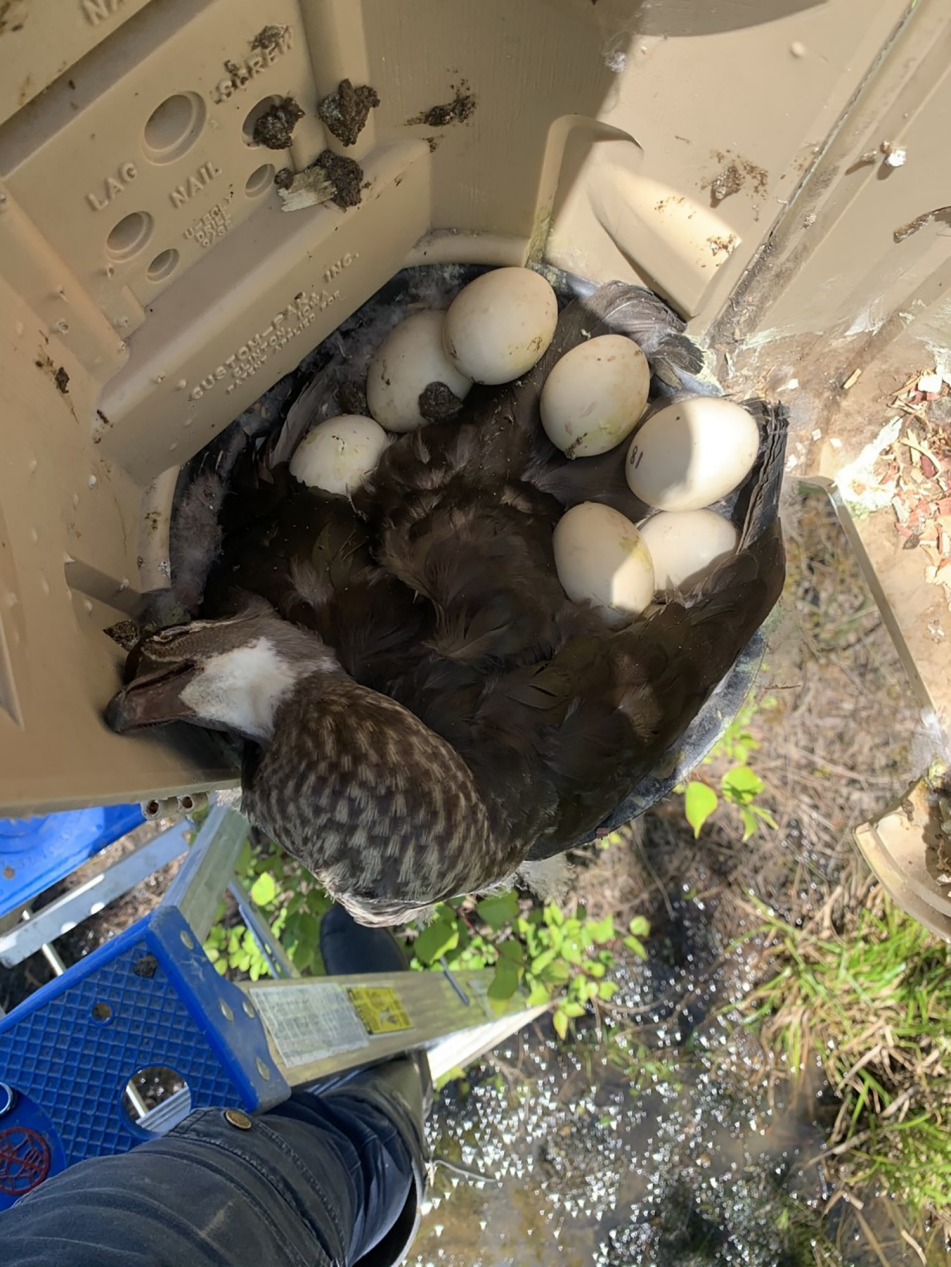
An image of the deceased wood duck hen and nest after the live hen was captured from nest box #N548 in Dover, Delaware on 4 May 2020.

Here, we report on the genetic relationship of two hen wood ducks observed to be simultaneously incubating a nest in Delaware, resulting in an agonistic interaction and subsequent death of one of the incubating females. A total of 239 wood duck artificial nest boxes–often used to assure reproductive success of local wood duck populations [9]–are annually monitored in central Delaware from early March to the end of July. During this time frame in 2020, monitoring crews identified two separate females incubating eggs in the same nest box on 1 May 2020 near Dover, Delaware (39.02575, -75.642972). One female flew from the box upon approach and another banded female (USGS 1165–71416) was found still incubating inside the box #N548. During a subsequent nest check on 4 May 2020, a live banded hen (USGS 1165–71439) was found incubating a nest beside the originating female (USGS 1165–71416) that was now deceased (Fig 1). The deceased hen was removed from the box on 4 May 2020. The live female continued to incubate for two days when all but one egg successfully hatched and the young fledged. Interestingly, in the previous 2019 nesting season, both females were captured and banded while using the same box (N548) in two separate nesting events. In 2019, the ultimately deceased female (USGS 1165–71416) was captured, aged (after second year), and banded while incubating. Her nest was the first established in box N548 and produced 12 ducklings from a clutch of 14 eggs on 16 April 2019. On 21 May 2019, the live female (USGS 1165–71439) was captured, aged (second year), and banded while incubating. This was the second nest established in box N548 in 2019 and consisted of 10 eggs and was determined abandoned by the female on 19 June 2019. Thus, both of these females displayed strong nest site philopatry, returning and using box N548 for a minimum of two breeding cycles.

We aimed to use molecular methods to determine the relatedness of the two females and the eggs found within the single nest box. Given the eagerness of the live female to complete incubation of the nest as if the clutch of eggs contained related genes, we proposed a hypothesis that the two females could have been closely related (i.e., sisters; [14]). An alternative hypothesis was the eggs of the single nest likely represent several maternal lineages (given that wood ducks are known to readily parasitize nests regardless of relatedness, [15]). Relatedness across samples was assessed by assaying the mitochondrial control region and hundreds of ddRAD-seq autosomal markers to establish the maternal and bi-parental lineages, respectively. For example, if the two incubating females are sisters, as well as for any eggs that are siblings, we expect them to harbor the same mtDNA haplotype and be assigned as full siblings based on nuclear variation. Finally, we assessed the sex of each to determine whether the parasitizing female was preferentially laying eggs of a particular sex [16–18].

## Materials and methods

### Nest sampling and DNA isolation

The deceased female was moved to a -20°C freezer when found on 4 May 2020. Once the young fledged and hens ceased using the nest box, monitoring crews immediately removed all eggshells, and the single inviable egg, and moved them to a -20°C freezer on 6 May 2020. Additionally, ~0.1 mLs of blood was drawn from the second live female on 6 May 2020 and immediately frozen at -20°C.

We attempted to extract DNA for the two adult females and 16 eggs using a Qiagen DNAeasy blood and tissue kit (Qiagen, Valencia, CA), and following manufacturer’s protocols. Extractions were quantified using a NanoDrop 2000 Spectrophotometer (Thermo Fisher Scientific Inc.), and levels of genome fragmentation visualized on a 1% agarose gel. To ensure success of dddRAD-seq libraries, samples with a minimum concentration of 0.02 μg/μl and a visible high molecular weight band were used [19].

### Sex identification

Sex across samples was based on the PCR amplification of CHD genes that are found on sex chromosomes, and which provide specific banding patterns when run on an agarose gel. In short, male (ZZ) and female (ZW) birds will show one or two bands, respectively. To do so, the published CHD1F/CHD1R primer pair [20] shown to be 100% efficient in Anseriformes [21] was used. PCR reactions comprised 2 μL of template DNA (≥10 ng/μl), 2x GoTaq Green Master Mix (Promega), and 1.0 nM of each primer, in a total volume of 15 μL and conducted using an Eppendorf Mastercycler (ep*gradient*) thermocycler. PCR conditions followed a touchdown protocol [19] and as outlined in Çakmak et al. [21] that included: DNA denaturation at 94°C for 4 minutes, followed by denaturation at 94°C for 30 seconds, annealing temperatures decreasing by 1°C per cycle from 57°C to 50°C, and followed by 30 cycles of 50°C for 45 seconds, an extension at 72°C for 45 seconds per cycle, and a final extension at 72°C for 5 minutes. Following, the full 15μL PCR mixture was loaded on a 3% agarose gel and run at a voltage of 110 for 45 minutes to visualize and sex each sample.

### ddRAD-seq library preparation, bioinformatics, & relatedness

Multiplexed ddRAD-seq fragment libraries followed protocols outlined in Lavretsky et al. [22]. In short, 10 U of Sbfl and EcoRI restriction enzymes were used to digest ~1 μg of gDNA. Subsequently, we ligated a unique combination of indices and barcodes to each samples to allow for multiplexing. Next, DNA ranging from 300 to 450 bp were excised through gel electrophoresis on a 2% agarose gel and purified using a MinElute Gel Extraction Kit (Qiagen, Valencia, CA). Selected DNA was then amplified using standard PCR with Phusion high-fidelity DNA polymerase (Thermo Scientific, Pittsburgh, PA). Products were purified using a 1.8x concentration of magnetic AMPure XP beads (Beckman Coulter, Inc., Indianapolis, IN), and quantified using a broad range assay on a Qubit (Thermo Scientific, Pittsburgh, PA). All samples were then pooled in equimolar concentrations, and 150 base pair, single‐end sequencing was completed on an Illumina HiSeq X at Novogenetics (Sacramento, CA). Raw Illumina reads are deposited in NCBI’s Sequence Read Archive (SRA; http://www.ncbi.nlm.nih.gov/sra; SRA data *PRJNA761429; BioSample Accession Numbers SAMN21332667—SAMN21332676*).

Raw Illumina reads were de-multiplexed, processed, and de-novo assembled using the computational pipeline described by DaCosta and Sorenson ([23]; http://github.com/BU-RAD-seq/ddRAD-seq-Pipeline). In short, identical reads are first collapsed and filtered based on a Phred score < 20, followed by being clustered and concatenated with an–id setting of 0.85 using the UCLUST function in USEARCH v. 5 [24]. Loci are then aligned with MUSCLE v. 3 [25] and genotyped using the Python script RADGenotypes.py. Individual genotypes fell into four general categories: “missing” (no data), “good” (unambiguously genotyped), “low depth” (recovered data, but could not reliably score as homozygous or heterozygous because of low depth), and “flagged” (recovered heterozygous genotype, but with counts of major and other alleles below acceptable thresholds). We also considered any locus with <10x sequence coverage as missing.

Pair-wise sample nuclear relatedness was quantified using the program COLONY v. 2.0.6.5 [26]. COLONY implements full-pedigree likelihood methods to simultaneously infer sibling relationship and parentage among individuals using multi-locus genotype data. To ensure a robust nuclear dataset, relatedness was based on loci with no missingness and ≤ 3 flagged genotypes across samples. Additionally, we used BLASTN v. 2 [27] to assign BLAST ID scores to autosomal chromosomes based on the published mallard (*Anas platyrhynchos*) genome ([28]; SRA BioProject PRJNA554956 & BioSample SAMN12287667) and using default settings across parameters. Only those to autosomal chromosomes with perfect BLAST ID scores were analyzed. In the end, COLONY analyses were based on ddRAD-seq autosomal bi-allelic SNPs with no missing data and a minimum allele frequency of 0.5 across samples. To reduce the risks of type I error, we only reported parental, full-sibling, and half-sibling dyads with pairwise relatedness estimates that were greater than 0.2 [29]. Analyses were based on ten independent runs using the “very long” algorithm, monitor intermediate results every second, and with assumptions that include polygamous mating systems for males and females, updating allele frequencies by accounting for the inferred relationship, and sibship scaling. We also used a probability of 1 that one of the females represented a candidate mother.

Finally, the same 420 ddRAD-seq SNPs was also used to visualize nuclear structure among samples by performing a principal component analysis (PCA) using the “dudi.pca” function in the R package Adegenet [30].

### Mitochondrial DNA sequencing and alignment

Primers L78 and H774 were used to PCR amplify and sequence 625 bp of the mtDNA control region [31, 32] following Sanger Sequencing methods described in Lavretsky et al. [33]. Resulting PCR products were sequenced using the L78 primer on a 3130XL Genetic Analyzer at the University of Texas at El Paso, Border Biomedical Research Center’s Genomic Analysis Core Facility. We aligned and edited sequences using Sequencher v. 4.8 (Gene Codes, Inc). All sequences are deposited in GenBank (*accession numbers OK083641—OK083657*). In addition to simply comparing sequences to determine similarity, we visualized maternal structure via a haplotype network reconstructed in the program Network v. 5 [34].

## Results

Sufficient DNA quality and quantity was achieved across samples. Mitochondrial DNA and nuclear ddRAD-seq loci were successfully sequenced for both females. Of the 16 eggs, two failed to amplify the CHD genes used for sexing (87.5% success rate), one failed to amplify for mtDNA (93.75% success rate), and only eight (50% success rate) successfully amplified wood duck ddRAD-seq nuclear loci. Note that we were able to construct ddRAD-seq libraries that yielded >1 million reads per egg sample (Table 1); however, only eight of these had overlapping wood duck sequences, while the remaining eggs provided deeply sequenced genes of alternative origin (i.e., non-duck) as none of them had BLAST identity to the mallard genome.

**Table 1:** Sample information including total number of Illumina sequencing reads, sex identification, mitochondrial (mtDNA) haplotype ID, and inferred maternal and paternal lineages using nuclear ddRAD-seq SNPs in the program COLONY.

Sample ID	Individual	# Raw Illumina Sequences	CHD Based SEX	mtDNA Haplotype	COLONY Inferred Father ID	COLONY Inferred Mother ID
WODU.F1	FEMALE (Dead)	1,737,030	Female	HAP.F1	Unrelated Female 1	Unrelated Female 1
WODU.F2	FEMALE (Alive)	1,925,373	Female	HAP.F2	Unrelated Female 2	Unrelated Female 2
WODU.E3	EGG.1	1,519,864	Male	HAP.F2	FAILED	FAILED
WODU.E4	EGG.2	1,512,280	Male	HAP.F2	FAILED	FAILED
WODU.E5	EGG.3	34,741	FAILED	HAP.F1	FAILED	FAILED
WODU.E6	EGG.4	682,156	Female	HAP.F1	FAILED	FAILED
WODU.E7	EGG.5	1,031,596	Male	HAP.F1	FAILED	FAILED
WODU.E8	EGG.6	1,325,314	Male	HAP.F2	FAILED	FAILED
WODU.E9	EGG.7	356,271	Male	HAP.F1	1*	WODU.F1
WODU.E10	EGG.8	977,599	Male	HAP.F1	1*	WODU.F1
WODU.E11	EGG.9	77,014	Male	HAP.F1	1*	WODU.F1
WODU.E12	EGG.10	478,213	Male	HAP.F2	2*	WODU.F2
WODU.E13	EGG.11	10,165	Female	FAILED	3*	1*
WODU.E14	EGG.12	1,833,434	Male	HAP.F3	FAILED	FAILED
WODU.E15	EGG.13	458,059	Female	HAP.F1	1*	WODU.F1
WODU.E16	EGG.14	1,054,099	Male	HAP.F1	4*	WODU.F1
WODU.E17	EGG.15	266,453	Female	HAP.F1	1*	WODU.F1
WODU.E18	EGG.16^1^	1,905,732	FAILED	HAP.F1	FAILED	FAILED

^1^: Inviable Egg

*: Inferred lineage.

In the end, we successfully sequenced 660 base pairs (bp) of the mtDNA control region across 17 samples, and 222 ddRAD-seq autosomal loci (31,287 bp; 649 polymorphisms) across 10 samples that met our coverage and missing data criteria. From a total of 7,340,276 raw Illumina sequence reads (range = 10,165–1,925,373), we attained a median average depth of 196 (median range = 13–897) across the samples for which we attained ddRAD-seq loci (Table 1); final per sample depths corresponded with their respective starting raw sequence number.

**Fig 2:**
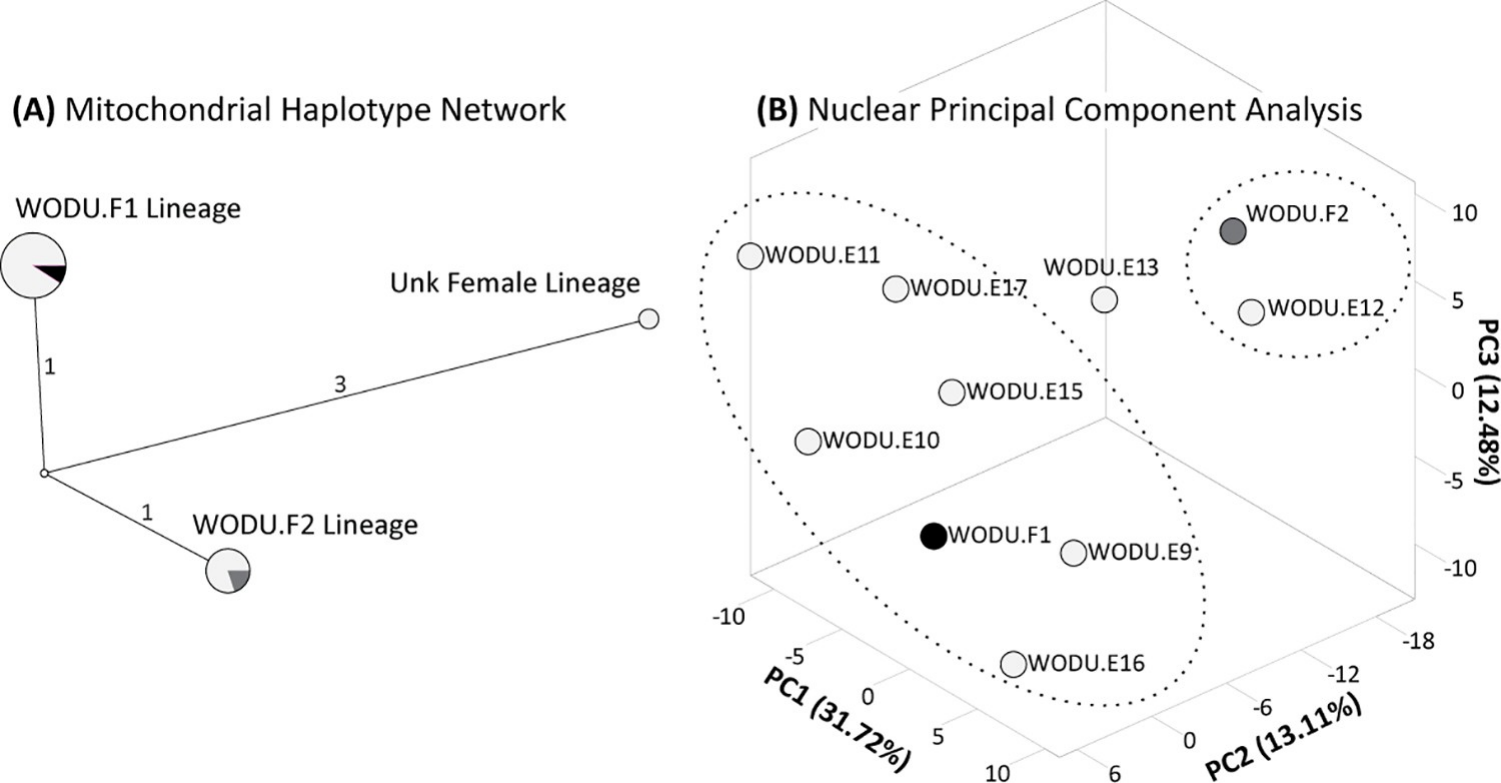
**(A)** A haplotype network reconstructed form 660 base-pairs of the mitochondrial control region across the two incubating females and 15 eggs (Table 1). Circles denote different haplotypes, including the two female’s lineages, and circle size is proportionate to the number of samples represented within the haplotype. The total number of mutations separating haplotypes are also provided. **(B)** Plotting of the first three principal components from our PCA of 420 ddRAD-seq nuclear SNPs. Once again, the two females are color coded, and family groups denoted by dotted circles.

First, we found three mtDNA lineages that were distinguished by several substitutions each (Fig 2A). Importantly, the two incubating females did not share the same maternal lineage as their mtDNA sequences were different by two substitutions. When multiplying the mtDNA mutation rate (4.8 × 10^−8^ substitutions/site/year; [35] by 660 bp of sequenced mtDNA control region gets us a rate of 3.2 × 10^−5^ substitutions/year; which translates to ~1 substitution (i.e., a mutation that is maintained in the population) every~32,000 years. Given that the three recovered mtDNA haplogroups are separated by either 4 (Unk Female vs F1 and F2) or 2 (F1 vs F2) mutations, they can be considered fairly diverged. Consequently, the three mothers indeed represent unique maternal lineages within the surveyed population. Among the 15 eggs for which we obtained mtDNA, eleven (69% of the clutch, including the one inviable egg) were assigned to the deceased incubating female and four (27% of the clutch) were assigned to the live incubating female. Interestingly, a single egg (6.7% of the clutch) was found to have a very unique mtDNA haplotype that was distinguished by four substitutions and unshared with either of the two known females or any other egg, suggesting this egg was the result of a parasitizing event by another unrelated female. Next, clutch composition for the first female included five males, three females, and one that did not amplify, whereas the live and unknown females only had males (Table 1).

**Fig 3:**
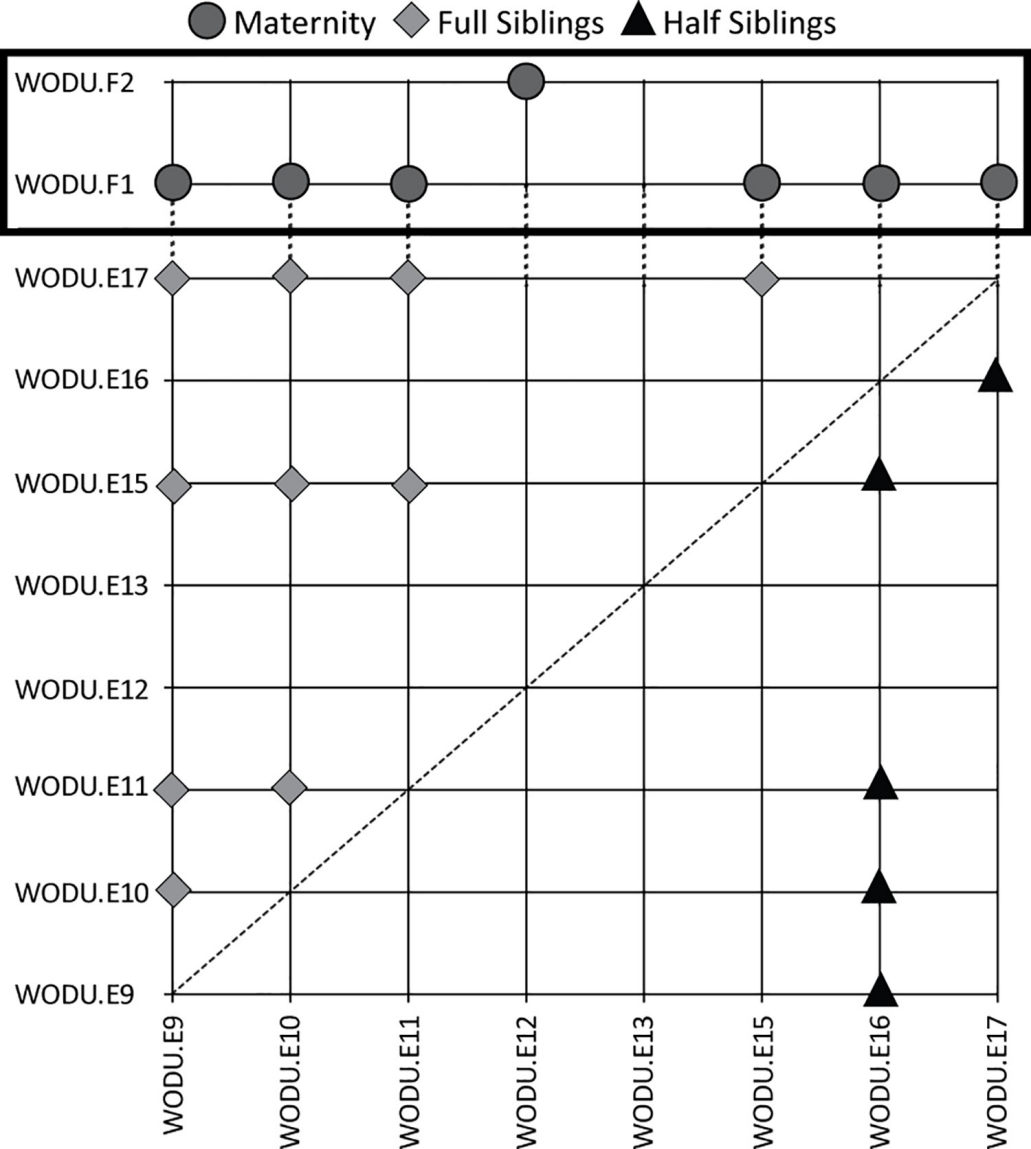
Maternity (boxed) and sibling (full and half) relationships across available samples (Table 1) based on a COLONY analysis of 420 ddRAD-seq nuclear SNPs.

Next, a total of 420 ddRAD-seq autosomal SNPs that met our coverage and minimum allele frequency requirements were used to quantify relatedness among samples in the program COLONY (Fig 3). Corresponding with mtDNA, the two incubating females were identified as unrelated at nuclear DNA as well. Next, among the eggs for which we attained sufficient ddRAD sequence data, six carried the mtDNA lineage of the deceased female, only one with the mtDNA lineage of the live female, and another that represented an unknown mtDNA lineage (i.e., failed to amplify for mtDNA; Table 1). For the deceased female, we inferred full sibling relationship between five of the eggs. Interestingly, one of the deceased female’s eggs showed a half sibling relationship to the other five full siblings (Fig 3), and for which COLONY inferred a distinct paternal lineage (Table 1). The single egg sequenced that carried the live female’s mtDNA was indeed assigned as the maternal lineage, and for which COLONY also inferred a distinct paternal lineage (Table 1). Finally, for the egg that failed to amplify for mtDNA but was identified as female, COLONY inferred distinct maternal and paternal lineages; which suggests that the egg may represent parasitizing event from a second unrelated female, for a total of four females contributing to the nest.

## Discussion

Through the molecular assessment of a single wood duck egg clutch in an artificial nest box and the two females found incubating those eggs, we conclude that (1) the two incubating females were entirely unrelated, (2) the single clutch is in fact represented by a minimum of four unrelated females, and (3) a single female can lay eggs sired by different males (Table 1). First, the un-relatedness among the parasitizing females is concordant with previous studies [7, 12, 15], but conflicting with others [13], which further supports that the probability of a nest being parasitized is likely population-specific. We posit the cause for the apparent competition and aggression among these unrelated females is likely due to limitations in preferred nesting sites as suggested by high occupancy rates and prevalence of nests with large egg dumps among our Delaware nest boxes.

Next, sibship analysis revealed that prior to her demise, the deceased female laid an egg determined as a half-sibling to the rest of her eggs, and thus making this a multi-paternal clutch. Although, the majority of eggs being identified as full siblings, and thus having the same father supports this female having a primary male with which she was likely pair-bonded with, the additional paternal lineage suggest this female must have engaged in extra-pair copulation (Fig 2). Extra-pair copulation (EPC) is a reproductive strategy now documented in 55 species of waterfowl, including the genera *Aix* [36]. EPC is often instigated by males of a population [37], and though incidences of such events can be high (e.g., ~50%; [38]), such events rarely lead to offspring (i.e., ~5% of all EPC events; [38, 39]). The generally low success of EPC events is the likely byproduct of the complex genetilia systems (i.e., reversed corkscrew with dead-ends; [40]) of waterfowl that allow females to control sperm fertilization [40, 41]. Although, behavioral observations have suggested that EPC occur among wood ducks [9], we provide the first genetic data that in fact such actions not only occur but can result in multi-paternal clutches. Unfortunately, we were unable to genetically vet all of the eggs to determine the true proportion of eggs sired by this secondary father. Moreover, the capacity of females to control the success of EPCs, and specifically, whether secondary paternal egg fertilization is random chance or female controlled is still not entirely known [41]. The ability for almost continuous and population-level monitoring due to their cavity-nesting behavior, wood ducks provide a unique system that can shed light into the evolutionary and adaptive consequences of female nest parasitism and male EPCs. In particular, future studies into understanding how these two life-history traits impact population dynamics can take advantage of the capacity and reducing costs of next-generation sequencing methods to attain fine-scale understanding of clutch compositions across time and space.

Genetic methods provide novel insight into nesting ecology of species that are often undetectable with other practices [7, 12]. For instance, traditional wood duck box monitoring typically uses clutch size or egg deposition rates to determine parasitic nests (e.g., [11]. However, these methods may underestimate parasitic rates [7], as there is overlap between clutch sizes of parasitized and non-parasitized nests. Additionally, even if nests are checked daily, a parasitic egg may go undetected if the host skips laying for one day. The use of high through-put genetic techniques applied to nest materials after hatch or nest failure reduces the uncertainty of both previously mentioned methods [12]. Behavioral observations are another technique used to study parasitic behavior (i.e., [7]), which are time consuming and resource intensive. Collection of genetic samples, especially noninvasive sampling of chorioallantoic membranes can provide a way to obtain more information with fewer resources and less disturbance. However, proper collection and storage of samples is important to ensure maximum success of partial genome sequencing methods which require large amounts of quality DNA. In general, given that the eggs were sampled sometime after hatching, the hot and humid environment of a nest box clearly provided an ideal environment for bacterial growth as observed here, and thus, may be the cause of our 50% success rate that is comparable to other similar studies [42]. To increase success, samples ideally should be flash frozen and stored at -80°C as soon as possible [43]. Nevertheless, coupling nuclear, mtDNA, and genetic-based sex identification proved to be successful in determining relation of competing females, and provided a more complete picture of the nesting ecology of Delaware’s wood ducks. Overall, with decreasing costs and increasing effectiveness, genomic methods have the potential to provide important insights into more complex ecological and evolutionary tactics of such populations.
